# Genomic Insight Into the Population Admixture History of Tungusic-Speaking Manchu People in Northeast China

**DOI:** 10.3389/fgene.2021.754492

**Published:** 2021-09-30

**Authors:** Xianpeng Zhang, Guanglin He, Wenhui Li, Yunfeng Wang, Xin Li, Ying Chen, Quanying Qu, Ying Wang, Huanjiu Xi, Chuan-Chao Wang, Youfeng Wen

**Affiliations:** ^1^Institute of Biological Anthropology, Jinzhou Medical University, Jinzhou, China; ^2^State Key Laboratory of Cellular Stress Biology, National Institute for Data Science in Health and Medicine, School of Life Sciences, Xiamen University, Xiamen, China; ^3^Department of Anthropology and Ethnology, Institute of Anthropology, School of Sociology and Anthropology, Xiamen University, Xiamen, China; ^4^State Key Laboratory of Marine Environmental Science, Xiamen University, Xiamen, China; ^5^School of Humanities, Nanyang Technological University, Singapore, Singapore; ^6^Xinbin Manchu Autonomous County People’s Hospital, Fushun, China

**Keywords:** East Asia, genetic admixture, genetic structure, population genetics, population history

## Abstract

Manchu is the third-largest ethnic minority in China and has the largest population size among the Tungusic-speaking groups. However, the genetic origin and admixture history of the Manchu people are far from clear due to the sparse sampling and a limited number of markers genotyped. Here, we provided the first batch of genome-wide data of genotyping approximate 700,000 single-nucleotide polymorphisms (SNPs) in 93 Manchu individuals collected from northeast China. We merged the newly generated data with data of publicly available modern and ancient East Asians to comprehensively characterize the genetic diversity and fine-scale population structure, as well as explore the genetic origin and admixture history of northern Chinese Manchus. We applied both descriptive methods of ADMIXTURE, fineSTRUCTURE, *F*_*ST*_, TreeMix, identity by decedent (IBD), principal component analysis (PCA), and qualitative *f*-statistics (*f*_3_, *f*_4_, qpAdm, and qpWave). We found that Liaoning Manchus have a close genetic relationship and significant admixture signal with northern Han Chinese, which is in line with the cluster patterns in the haplotype-based results. Additionally, the qpAdm-based admixture models showed that modern Manchu people were formed as major ancestry related to Yellow River farmers and minor ancestry linked to ancient populations from Amur River Bain, or others. In summary, the northeastern Chinese Manchu people in Liaoning were an exception to the coherent genetic structure of Tungusic-speaking populations, probably due to the large-scale population migrations and genetic admixtures in the past few hundred years.

## Introduction

The Manchu is the third-largest ethnic minority with a population size of over 10 million in China; and they mainly live in Liaoning, Hebei, Heilongjiang, and other northern provinces. Liaoning Province was the traditional homeland of the Manchu people, and the first capital of the Qing Dynasty was located there. There are more than 5 million Manchus in Liaoning, accounting for more than 50% of the total population of Manchu. The term ‘‘Manchu’’ can be dated back to the 16th century, but the history of Manchu can be traced back further. According to early historic records, the Manchu ancestors were known as Donghu, a tribal confederation of nomadic people that was first recorded from the seventh century BCE. After then, the history of Manchu had been associated with many ancient tribes that once lived in this region during different periods, such as Sushen, Yilou, Wuji, and Mohe. In the late historic period, there are two dynasties associated with the Manchus: the first one was Jin Dynasty^1^ (1115--1234 AD) founded by Jurchen, and the second was the Qing Dynasty^2^ (1636–1912 AD) founded by ancient Manchu people. After the Jin Dynasty was annihilated by the Mongols in 1234 AD, the surviving Jurchen people gradually developed into Manchu. When Manchu regained control of Manchuria, they moved further south and gradually controlled all sections of China, and they were suggested to have left detectable genetic imprints on the modern north and northeast Chinese, especially in the Qing Dynasty (Xue et al., 2005; Zhao et al., 2011; He and Guo, 2013).

Previous genetic studies of the Manchu population were predominantly based on very limited numbers of forensic markers, such as short tandem repeats (STRs) and single-nucleotide polymorphisms (SNPs) on the autosome (Liu et al., 2013), Y-chromosome (He and Guo, 2013), X-chromosome (Xing et al., 2019), and mtDNA (Zhao et al., 2011). The autosomal STR study of Manchu suggested that there were only small genetic distances between the Liaoning Manchus and Qinghai and Liaoning Hans (Liu et al., 2013). From a paternal Y chromosomal perspective, there were no significant differences in the haplotype composition between Liaoning Manchus and Northern Hans or Chinese Mongolians, and Manchus displayed a very typical East Asian affinity. Besides, there were only minor differences between Manchus and East Asian populations such as the Southern Han population, Chinese Korean, Japanese, and South Korean (He and Guo, 2013); Manchus shared similar Y-haplotypes with Xibe, Outer Mongolians, Inner Mongolians, Ewenki, Oroqen, and Hezhen (Xue et al., 2005). The investigation of the Y-chromosomal profile of the Aisin Gioro clan who are the Qing Dynasty nobility found that Manchus might be descendants of ancient populations in the Transbaikal region (Yan et al., 2015; Wei et al., 2017; Wang et al., 2019). The X-chromosomal profile further showed that there was an affinity between the Liaoning Manchus and Liaoning Koreans and Hans from Henan and Shanghai (Xing et al., 2019). From a maternal mtDNA perspective, Liaoning Manchus displayed an admixture signal between northern and southern East Asians, and they had a close genetic affinity with neighboring populations, such as the Mongolians, Liaoning Hans, and Korean (Zhao et al., 2011). The genetic distances between the Manchus and the Altaic language-speaking populations such as Hezhen, Daur, and Oroqen were smaller than those of other language-speaking populations (Zhao et al., 2011; Liu et al., 2013; Xing et al., 2019). Most genetic investigations based on the high-density genome-wide genetic variations from non-Altaic people in East Asia have revealed a fine-scale genetic landscape of genetic diversity and population admixture among the populations from different-language families (He et al., 2021a,c; Liu et al., 2021a,b; Wang et al., 2021d; Yao et al., 2021). Besides, recent genome-wide studies among Altaic-speaking populations in Northeast Asia have also found differentiated genetic admixture profiles between northern and southern Altaic-speaking populations and eastern and western Mongolians (He et al., 2021b). The reconstructed demographic models using ancient genomes further showed the Tungusic people keep a strong genetic homogeneity within populations, and the type of Tungusic-dominant ancestry probably originated in a vast region from Mongolian Plateau to Amur River Basin about at least 16,000 years ago (He et al., 2021b; Mao et al., 2021). However, there are few genome-wide SNP data from Tungusic-speaking Manchu people reported so far.

Manchu language belongs to the Tungusic group of the Altaic language family, but previous genetic studies indicated that Manchus were genetically different from other Tungusic-speaking groups, which is probably due to the genetic influence from surrounding Han Chinese into Manchus (Xue et al., 2005; He and Guo, 2013). Chen et al. recently reported that Guizhou Manchus in southwest China had a strong genetic affinity with southern East Asians and found that Guizhou Manchus could be modeled as deriving a large proportion of southern ancestry related to Austronesian, Tai-Kadai, and Austroasiatic speakers, suggesting that Manchu gradually mixed with the southern natives along with their southward migration (Chen et al., 2021). Genetic diversity and genetic admixture scenarios of northern East Asian Altaic people were mainly collected from Mongolic and Tungusic people (Jeong et al., 2019; Wang et al., 2021b,c). The genetic structure, population origin, and admixture history of Manchus due to the paucity of genome-wide data from northeast China—the origin center of ancient Manchu people—are now far from clear. In this study, we reported for the first time the genome-wide SNP data of 93 Manchu individuals who have lived in the Xinbin Manchu Autonomous County (the location of Hetu Ala city), Liaoning Province in northeast China. Our aim was to comprehensively infer the genetic origin and explore the population admixture history of the Manchu people by coanalyzing both modern and ancient Eurasian genomes.

## Materials and Methods

### Sampling and Genotyping

**FIGURE 1:**
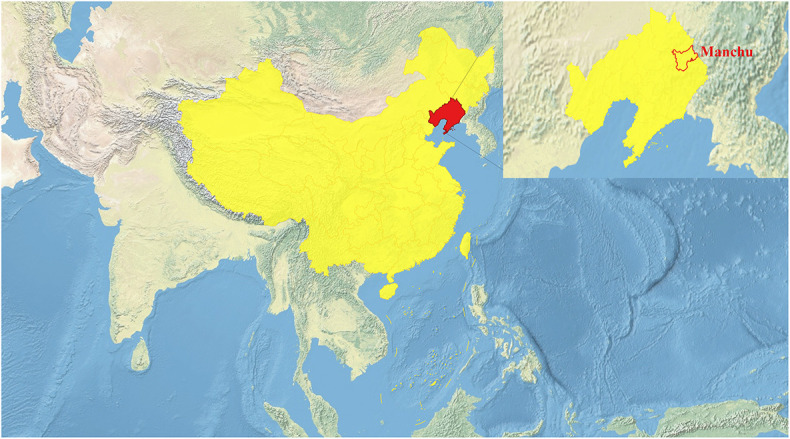
Map of the sampling location for this study (https://www.qgis.org/).

We collected a total of 93 peripheral blood samples from unrelated Manchu individuals aged over 58 in different villages in the Xinbin Manchu Autonomous County, Liaoning Province, northeast China (Figure 1). These samples were collected randomly from unrelated participants whose parents and grandparents are indigenous people and have a non-consanguineous marriage of the same ethnical group for at least three generations. The ethnicities of all participates were used as their self-declaration based on their family migration history and corresponding family records. Our study and sample collection were reviewed and approved by the Ethics Committee of Jinzhou Medical University (JZMULL2021010) and followed the recommendations provided by the revised Declaration of Helsinki of 2000. Verbal and written informed consent was obtained from all participants. We used the Infinium^®^ Global Screening Array (GSA) covering 659,509 SNPs to genotype targeted SNPs in Manchus. Genotype calling was carried out following the default parameters. Raw data were initially filtered using PLINK 1.9 (Purcell et al., 2007) based on our predefined threshold of the genotyping success rate, missing site rates, minor allele frequency, and Hardy–Weinberg equilibrium (−maf:0.01, −hwe:0.0001, −mind:0.01, and −geno:0.01). A final dataset with 293,307 SNPs was used to perform the following population genetic analysis.

### Data Merging

We merged our newly genotyped data with previously published modern and ancient population data from the Affymetrix Human Origins (HO) Array dataset (Wall and Yoshihara Caldeira Brandt, 2016) and the 1240K dataset from the Allen Ancient DNA Resource (AADR).^3^ The included modern genome-wide SNP reference data were collected from nine language families or groups (Tungusic, Mongolic, Turkic, Sinitic, Tibeto-Burman, Austronesian, Austroasiatic, Tai-Kadai, and Hmong-Mien) in China, South Siberia, and Southeast Asia (Patterson et al., 2012; Lipson et al., 2018; Liu et al., 2020; Kutanan et al., 2021; Wang et al., 2021a). Ancient reference populations were also collected from China and surrounding countries (Ning et al., 2020; Yang et al., 2020; Wang et al., 2021a). We also included recently published population data from the Guizhou Manchu people (Chen et al., 2021). Finally, two combined datasets covering 44,476 SNPs in the merged HO set and 127,435 SNPs in the merged 1240K set were used in the subsequent analysis.

### Principal Component Analysis and Admixture

Principal component analysis (PCA) was carried out using *smartpca*, part of the EIGENSOFT package (Patterson et al., 2006). The additional parameter of the lsqproject was set as YES (lsqproject: YES), and other parameters were used as the default setting. A total of 70 present-day and ancient worldwide populations were selected for PCA. Ancient people were projected onto the background of modern genetic variations. The PCA result was plotted by R software.^4^ We applied ADMIXTURE (Alexander et al., 2009) to conduct the model-based clustering analysis based on the merged HO dataset, which consists of 1,385 individuals from 89 populations. Prior to the analysis, we pruned SNPs in strong linkage disequilibrium with each other using PLINK 1.9 (Purcell et al., 2007) with the parameters “-indep-pairwise 200 25 0.4.” We run ADMIXTURE with the K values (number of assumed ancestral sources) ranging from 2 to 20. An optimal value of K was selected using 10-fold cross-validation errors, which is shown in Supplementary Table 1. The results of admixture analysis were visualized using AncestryPainter (Feng et al., 2018).

### *F*_*ST*_ Analyses

We calculated pairwise *F*_*ST*_ genetic distance between Liaoning Manchu and other included modern and ancient reference populations using *smartpca* of EIGENSOFT package (Patterson et al., 2006) (fstonly: YES, fsthiprecision: YES).

### *f*-Statistics

All *f-*statistics were calculated using ADMIXTOOLS (Patterson et al., 2012). We computed outgroup *f*_3_-statistics in the form of *f*_3_ (Manchu_Liaoning, X; Mbuti) to examine the shared genetic drift between Liaoning Manchus and non-African reference populations. Central African of Mbuti was used as the outgroup. We also used the *f*_3_-test in the form of *f*_3_ (source1, source2; Manchu_Liaoning) to formally test whether there was an admixture signature in Liaoning Manchus with different source pairs from modern and ancient eastern Eurasians. Negative values with the absolute Z-score of less than −3 indicated the included two source-related populations may be the plausible ancestral sources. We applied the *f*_4_-test in the forms of *f*_4_ (X, Y; Manchu_Liaoning, Mbuti) and *f*_4_ (X, Manchu_Liaoning; Y, Mbuti) to estimate the differentiated allele sharing between Liaoning Manchus and other representative sources compared with focused comparative subjects, where X and Y are the included ancient and present-day populations. The tested form *f*_4_ (X, Y; Manchu_Liaoning, Mbuti) was designed to examine the differentiated genetic ancestry between Manchus and X or Y, in which significantly negative values indicate more shared alleles between Y and Manchus compared with X, significantly positive values indicated more shared alleles between X and Manchus relative to Y, and nonsignificant values (Z-scores ranged from −3 to 3) indicated that X and Y formed one clade compared with the Manchus (Patterson et al., 2012).

### *qpAdm* and *qpWave*

We used formal tests of the *qpWave*/*qpAdm* programs in ADMIXTOOLS (Patterson et al., 2012) to determine the minimum number of streams of potential source populations contributing to the tested populations and also estimate the admixture proportions. We used the following eight outgroups including Atayal, Mbuti, Papuan, French, DevilsCave, Jomon, Malaysia_LN, and Tianyuan, which are unlikely to have been affected by recent gene flow with proposed ancestral sources and might be differentially related to the tested populations. We chose the included outgroups as the distant outgroups to dissect the northern and southern East Asian ancestries that participated in the formation of modern Manchus based on recent modern and ancient genetic studies (Chen et al., 2019; He et al., 2020; Wang et al., 2021a).

### TreeMix and ALDER

To explore the genetic relationship between the Manchu population and other references East Asians, we used the TreeMix v1.13 (Pickrell and Pritchard, 2012) program to construct the maximum likelihood trees with variable predefined mixture events. The level and time of admixture events were estimated by using ALDER v.1.0.3 (Wu, 2020).

### Identity by Decedent and Chromosome Painting

We used shapeit v2 to phase successive SNPs into haplotype data and following used refined identity by decedent (IBD) to calculate the pairwise shared IBD (Browning and Browning, 2011). ChromoPainter (Lawson et al., 2012) and fineSTRUCTURE (Lawson et al., 2012) were further used to explore the fine-scale population structure based on the sharing haplotypes.

### Y Chromosomal and mtDNA Lineages

The mtDNA haplogroups were classified using HaploGrep2 (Weissensteiner et al., 2016) based on PhyloTree17^5^, and we used in-house scripts to assign the Y-chromosomal paternal lineage following the basic regulations reaccommodated via the International Society of Genetic Genealogy (2018)^6^.

## Results

### Principal Component Analysis and Admixture

**FIGURE 2:**
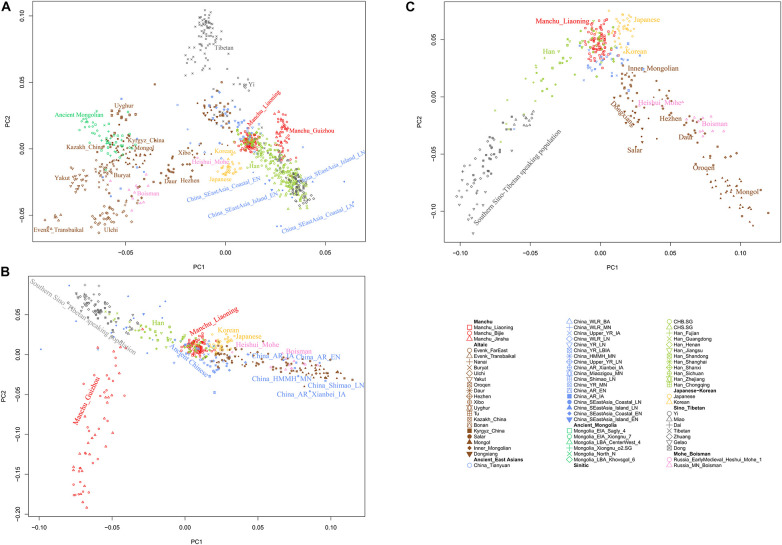
Patterns of genetic relationship among East Asian populations and Liaoning Manchus inferred from the principal component analysis **(A–C)**. Modern populations were color-coded on the basis of their language family categories, and different shapes represented different populations. All ancient populations were projected onto it.

To understand the general pattern of the genetic structure of Liaoning Manchus, we first performed PCA to explore the two-dimensional genetic relationship between Manchus and other reference eastern Eurasians. We found that the observed genetic clusters were consistent with the geographic and linguistic categories within East Asia (Figure 2). We observed in the PCA that Liaoning Manchus are genetically different with Turkic-, Tungusic-, and Mongolic-speaking groups in northwest China, Mongolia, and Siberia. Liaoning Manchus clustered closely to northern Han Chinese from Shanxi, Shandong, and Henan Provinces and also to Neolithic northern East Asians from the Yellow River and Western Liao River Basins.

**FIGURE 3:**
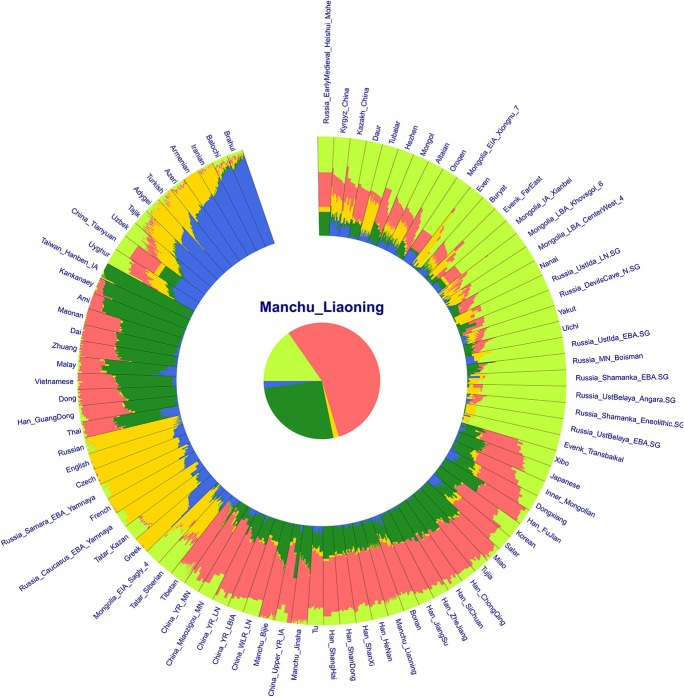
Results of model-based ADMIXTURE clustering analysis. Clustering patterns were visualized with the predefined ancestral sources at *K* = 5. All of these ancestral components were revealed by different colors.

**FIGURE 4:**
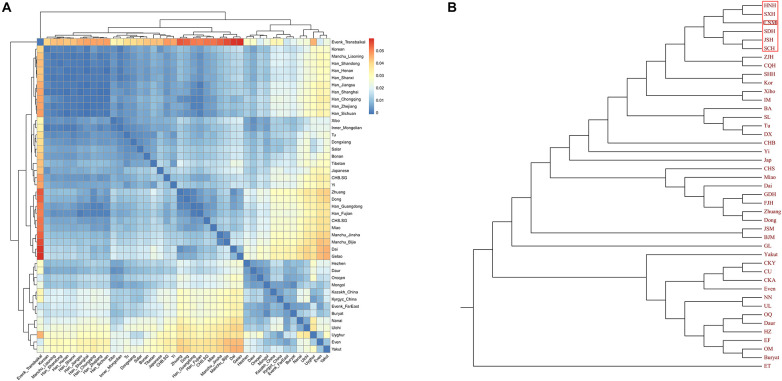
Results of pairwise *F*_*ST*_ among East Asian populations. The dark blue color in panel **(A)** reveals a close genetic distance between two different populations, and panel **(B)** displays the phylogenetic tree based on *F*_*ST*_ values.

In the model-based ADMIXTURE clustering analysis, we used cross-validation to identify an “optimal” number of clusters. We found the lowest CV error at *K* = 5 (Supplementary Table 1). At *K* = 5 (Figure 3), we observed there were three components of light green, dark green, and pink color reaching high proportions in Liaoning Manchus. The light green ancestry was enriched in the Tungusic people and ancient populations from the Baikal Lake region. Dark green ancestry with maximum proportion was observed in the southern Chinese populations and Southeast Asians, especially in Taiwan Hanben people. Pink ancestry was maximized in the Yellow River millet farmers. Therefore, Manchus had ancestry related to northeast Asians, Yellow River farmers, and southern East Asians. Similar genetic profiles were observed in the northern Han Chinese, suggesting a close genetic relationship between Manchu and northern Han. In pairwise *F*_*ST*_ analysis, we observed a similar pattern that Liaoning Manchus had the smallest genetic distance with Han Chinese in northern China such as in Shandong, Henan, and Shanxi (Figure 4 and Supplementary Table 2).

### *f*_3_- and *f*_4_-Statistics

**FIGURE 5:**
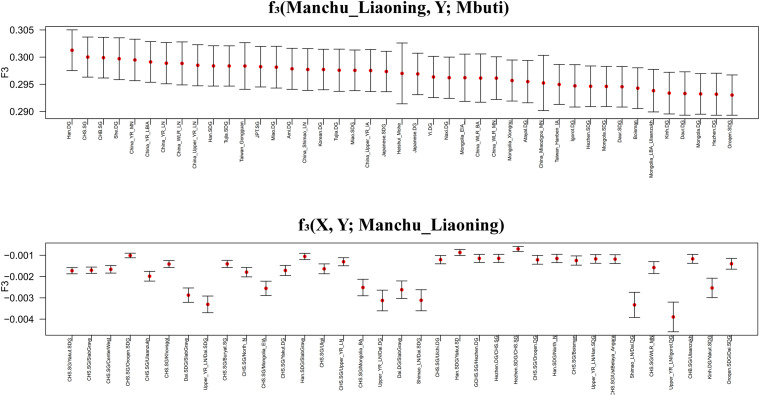
Shared genetic drift estimated via *f*_3_-statistics in different testing forms based on the merged 1240K dataset focused on Liaoning Manchus. Bar denotes three times of the error bar.

To further investigate the genetic origin and admixture of Liaoning Manchus, we performed outgroup-*f*_3_ and admixture-*f*_3_ statistics to measure allele sharing and detect admixture signals. In outgroup-*f*_3_ (Manchu_Liaoning, Y; Mbuti) (Figure 5 and Supplementary Table 3A), we found that Liaoning Manchus shared the most derived alleles with northern and southern Han Chinese, She, Tujia, Ami, Miao, Japanese, and Korean. When Y represented ancient individuals, Liaoning Manchus share more alleles with ancient East Asians from the Yellow River and Western Liao River Basins, consistent with the observed patterns in PCA and ADMIXTURE. We next used admixture-*f*_3_ statistics in the form of *f*_3_ (X, Y; Manchu_Liaoning) to detect possible admixture signatures, in which X and Y were modern or ancient populations that might be the candidate sources for modeling the admixture of Liaoning Manchus. We observed significant signals of admixture (Z < −5) in the Liaoning Manchus when using CHS or Dai as the southern source and present-day Oroqen, Yakut, Buryat, and Hezhen or ancient Mongolia populations as the northern ancestral source. We listed the Z < −5 in the Supplementary Material (Supplementary Table 3B).

We performed *f*_4_-statistics to explore genetic substructure between studied groups and other modern/ancient populations in the forms of *f*_4_ (X, Y; Manchu_Liaoning, Mbuti) and *f*_4_ (X, Manchu_Liaoning; Y, Mbuti) (Supplementary Table 4). When compared with modern Yakut, Tu, Buryat, and Thai populations, Liaoning Manchus shared more derived alleles with Han Chinese and Japanese. Compared with northern Tungusic, Mongolic, and Turkic people in Siberia, Manchu shared more alleles with the Tungusic- and Mongolic-speaking populations in China. When compared with the ancient populations from the Eurasian steppe, such as Yamnaya, EIA_Sagly, and EBA_Chemurchek, Liaoning Manchus shared more derived alleles with ancient populations from Yellow River Basin and West Liao River Basin, Boisman, and some ancient Mongolian such as EIA_SlabGrave and LBA_CenterWest. To further explore the differentiated allele sharing status between Manchu and Han Chinese, we conducted *f*_4_ (Han, Manchu_Liaoning; X, Mbuti); and we found significant negative values when X was the Tungusic and Mongolic people, suggesting that Liaoning Manchus harbored more Tungusic-related ancestry than did Han Chinese (Supplementary Table 5A). We further observed significant negative *f*_4_ values in the form *f*_4_ (Mongolic/Tungusic populations, Manchu_Liaoning; southern modern East Asians, Mbuti), suggesting that Manchu people shared more alleles with southern East Asians compared with Tungusic and Mongolic people in the Amur River Region. We also observed significant negative values in *f*_4_ (Xianbei, Manchu_Liaoning; X, Mbuti) when X represented Han, Dai, Tujia, or other southern populations, suggesting that there was gene flow from the southern part of China into Manchu after the Xianbei period. When X was ancient Eurasians, Liaoning Manchus shared more derived alleles with populations from Yellow River Basin and West Liao River Basin (Supplementary Tables 5B–H).

### TreeMix and qpAdm

**FIGURE 6:**
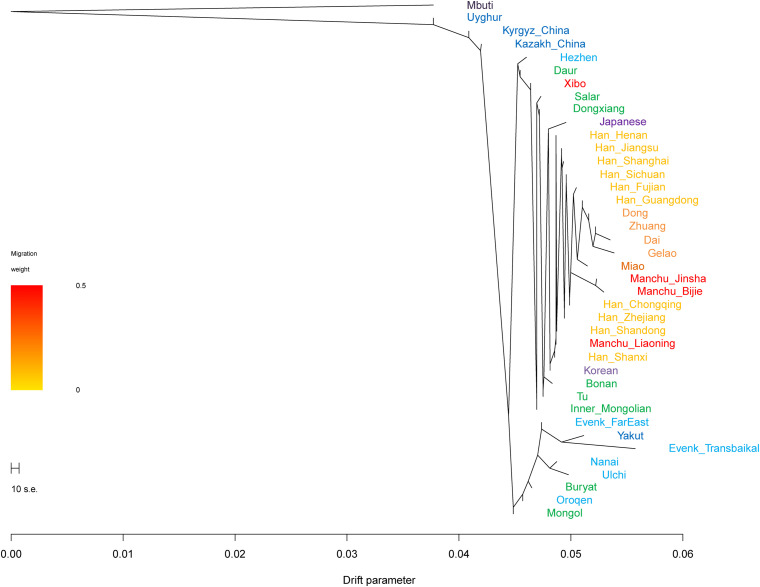
Phylogenetic relationships among Turkic, Tungusic, Mongolic, Tai-Kadai, and Hmong-Mien speakers. Mbuti population from central Africa was set as the root. Different populations were marked according to their linguistic affinity.

In the TreeMix analysis (Figure 6), we found that Tungusic-, Turkic-, and Mongolic-speaking groups in northern China tended to cluster together, but Liaoning Manchus clustered with northern Han Chinese and Guizhou Manchus clustered with southern Han Chinese and southern Tai-Kadai-speaking groups. The result was consistent with the patterns observed in the aforementioned PCA, ADMIXTURE, *F*_*ST*_, and *f*-statistics; Liaoning Manchus and Guizhou Manchus had experienced genetic influence from surrounding Han Chinese after they were separated from northern Tungusic- and Mongolic-speaking ancestors.

**FIGURE 7:**
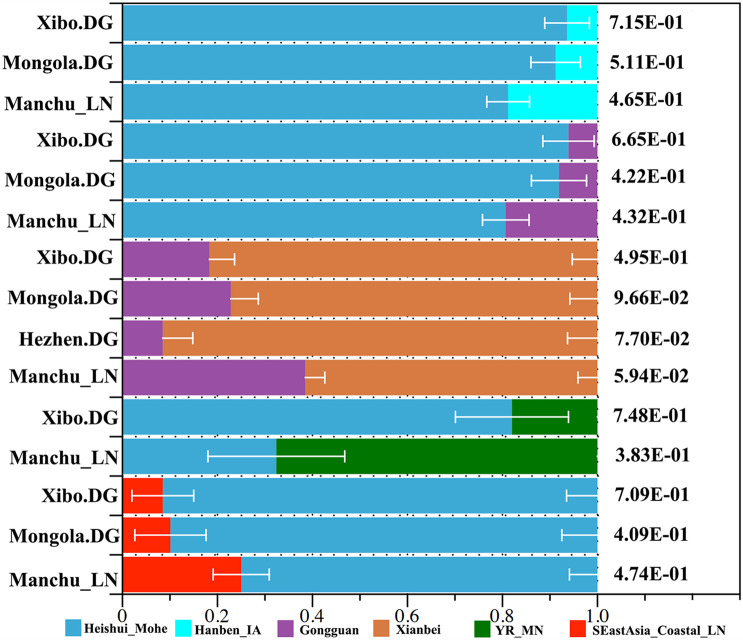
Model of ancient population admixture by *qpWave* and *qpAdm* method. All of these ancestral components were revealed by different colors.

We applied *qpWave* and *qpAdm* methods to further infer the possible ancestral populations and estimate the admixture proportions. We used the related available ancient northern populations (Heishui_Mohe, Boisman, Xiongnu, Xianbei, Yamnaya, Afanasievo, SlabGrave, Mongolia_North_N, CenterWest, MongunTaiga, Munkhkhairkhan, and Sagly) as the northern sources, used all available ancient populations (Miaozigou_MN, Shimao_LN, Upper_YR_IA, YR_MN, YR_LBIA, YR_LN, WLR_LN, and Upper_YR_LN) as the source of Yellow River sources, and used Iron Age Hanben (Hanben_IA) and Gongguan samples from Taiwan and Neolithic southern populations (SEastAsia_Coastal_EN, SEastAsia_Coastal_LN, SEastAsia_Island_LN, SEastAsia_Island_EN) as the southern sources. We observed that Manchus can be modeled as deriving 32.4% ancestry from Mohe people and the remaining ancestry from the farming-related ancient populations in the Yellow River Basin (Figure 7). When using Late Neolithic to Iron Age ancient southern populations (Hanben_IA, Gongguan and SEastAsia_Coastal_LN) as the southern sources, we found the proportion of northern ancestry (Heishui_Mohe and Xianbei) spanned from 61.5 to 81.2% (Figure 7). Compared with other Tungusic or Mongolic populations Hezhen, Mongolia, and Xibo, we observed Liaoning Manchus had derived more ancestry from farming-related populations in the Yellow River Basin and southern China and less ancestry from northern ancient groups, such as Heishui_Mohe and Xianbei.

### ALDER, Uniparental Haplogroups, and Chromosome Painting

We next used ALDER software to estimate when the admixture occurred. We tried different modern populations from the north and south of East Asia and Siberia as possible ancestral groups. We observed in most cases that the average time that the admixture occurred was around 500 AD (for example, 46.36 ± 20.93 generations) in the Southern and Northern Dynasties period.^7^ That period witnessed large-scale population migrations and admixtures due to the turbulence and war between Xianbei and Han Chinese.

We successfully identified 34 uniparental Y-chromosome lineages and 93 mtDNA lineages in Liaoning Manchus as shown in the Supplementary Material (Supplementary Table 6). We found that D4, A, and M8 were the dominant maternal lineages, and O2a2b1a2 was the dominant paternal lineage. Those paternal and maternal haplogroups are also dominant in Han Chinese, suggesting the possible gene flow from Han Chinese into the gene pool of Manchus.

**FIGURE 8:**
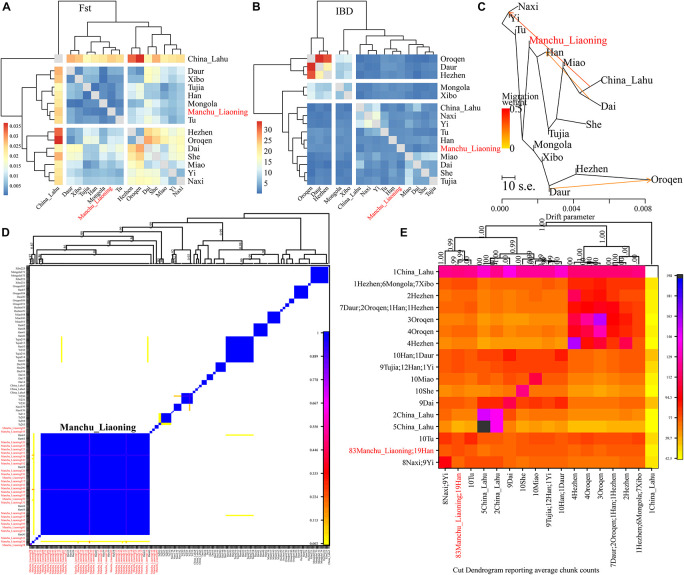
The deep population history of Manchus inferred from both sharing alleles and haplotypes. **(A,B)** Heatmap shows the pairwise *F*_*ST*_ genetic distance and identity by decedent (IBD). **(C)** The TreeMix-based phylogenetic tree shows the genetic relationship between Manchu people and other modern East Asians. **(D,E)** Clustering patterns based on the co-ancestry matrix.

Finally, to explore the fine-scale population structure of Manchu and other East Asians based on a higher-density dataset, we merged our data with 14 East Asian populations that were whole-genome sequenced in the Human Genome Diversity Project (HGDP) project. We performed the IBD-based clustering and calculated the pairwise *F*_*ST*_ genetic distances. We found that Manchus shared the most IBD with Han Chinese but had the smallest *F*_*ST*_ genetic distance with northern East Asians (Mongolia and Tu, Figures 8A,B). We have not detected the gene flow into Manchu people from our used plausible sources in TreeMix-based phylogeny with three gene flow events, but we found that Manchu clustered in between northern Mongolic and Tungusic people and southern Hans and other Hmong-Mien populations. Manchu clustered the closest with Han Chinese and Tujia (Figure 8C), which was further confirmed via the clustering patterns based on the fineSTRUCTURE-based chromosome painting patterns (Figures 8D,E).

## Discussion

Previous genetic studies have found that northern Han Chinese from Liaoning and Jilin Provinces had genetic admixture with Liaoning Manchus from the analysis of Y-chromosome and autosomal STRs (Yao and Wang, 2016; Xu et al., 2019). In this study, we also found that Liaoning Manchus had a significant admixture signal with Han Chinese, especially with the northern Han. For example, Manchus had more Sinitic-related ancestry component than other Altaic-speaking populations in ADMIXTURE. We proposed that Liaoning Manchus are an admixture of Han Chinese-related farming populations and local Tungusic-speaking populations in northeast Asia.

Manchus and their ancestors had lived in Manchuria for thousands of years before they moved south, invaded the Central Plains, and even established the Qing Dynasty, which also promoted gene flow between Manchus and Hans. The most well-known recent large-scale population migration event was the ‘‘Chuang Guandong’’^8^ (literally “Crashing into Guandong,” with Guandong being an older name for Manchuria). Northeastern China was the hometown of Manchus, and the first emperor of the Qing Dynasty was born there. With the establishment of the Qing Dynasty (see text footnote 2), Manchuria did not allow people who were non-Eight Banner to enter. Manchuria was vast and rich in material but sparsely populated. Therefore, many Han Chinese who lived in Hebei and Shandong Provinces left their hometown and went to Manchuria for survival because of various reasons such as multi-year natural disasters and shortage of food (Reardon-Anderson, 2000; Reardon-Anderson, 2005). Within the last 300 years, at least 30 million immigrants traveled far away over the mountains across the seas to northeast China. Han Chinese from Shandong, Hebei Province, and other regions lived together with the native Manchu population in northeast China. Until 1840, Han Chinese had filled up most of Manchuria’s cities and towns and left profound impacts on Manchu people in various aspects (Reardon-Anderson, 2000). For example, almost all Manchu people can speak Chinese; and in recent years, they had even abandoned the Manchu language. At the end of the Qing Dynasty, in order to consolidate its dominant position, the Qing government announced that the Manchus and Hans were one family and abandoned various restrictions on Han people to allow the Manchu nobility to marry Han people (Jones and Kuhn, 1978). However, intermarriages between Manchu people and Han people were in fact very common among ordinary people.

ALDER-based admixture time estimations revealed Mongolian and Even and Manchus have genetic admixture in ∼500 AD, which was the period of Southern and Northern Dynasties (see text footnote 6) (420–589 AD). It was a turbulent period of war and also a period of large-scale population admixture. At that time period, the Xianbei population controlled the Amur River Basin, Mongolian Plateau, and southern Siberia, and they became the largest ruling power in the North. In this study, we can also model Manchus deriving ancestry from the Xianbei people.

Previous studies have found strong associations between population genetic structure and linguistic similarity in Asia, and the populations from the same language group have a closer genetic affinity (Chen et al., 2019; He et al., 2020). Mongolic- and Tungusic-speaking populations were reported to be genetically similar (Ning et al., 2020; Wang et al., 2021a). In this study, we found that both Mongolic- and Tungusic-speaking populations have ancestry components related to ancient Mohe and Xianbei people. We proposed there were many cultural interactions and gene flows between Mongolic- and Tungusic-speaking populations.

## Conclusion

We reported the first research-based genome-wide SNP data of the Manchus from Liaoning Province. We used comprehensive population genetic analyses of PCA, ADMIXTURE, *qpAdm*, *qpWave*, *f-*statistic, *F*_*ST*_, ALDER, IBD, fineSTRUCTURE, and TreeMix to investigate the complex genetic history and dynamic admixture process of northern Chinese populations. Our previous study documented a long-term genetic continuity from Neolithic hunter-gatherers to present-day Tungusic-speaking people in northeast Asia. It is therefore believed that the Manchu people, being members of an old branch of the Tungusic, should have a consistent genetic profile with other Tungusic groups. However, we found that Liaoning Manchus have a close genetic relationship and significant admixture signal with Han Chinese. The Manchu population was an exception to the coherent genetic structure of Tungusic-speaking people, probably due to the large-scale population migrations and genetic admixtures in the past hundred years.

## Data Availability Statement

The datasets presented in this study can be found in online repositories. The names of the repository/repositories and accession number(s) can be found in the article/Supplementary Material.

## Ethics Statement

The studies involving human participants were reviewed and approved by Our study and sample collection were reviewed and approved by the Ethics Committee of Jinzhou Medical University (JZMULL2021010) and followed the recommendations provided by the revised Helsinki Declaration of 2000. The patients/participants provided their written informed consent to participate in this study.

## Author Contributions

YoW and C-CW designed the study. XZ and GH analyzed the data and wrote the manuscript. WL, YuW, YiW, and HX carried out the sample collection. XL, YC, and QQ conducted the experiment. All authors contributed to the article and approved the submitted version.

## Conflict of Interest

The authors declare that the research was conducted in the absence of any commercial or financial relationships that could be construed as a potential conflict of interest.

## Publisher’s Note

All claims expressed in this article are solely those of the authors and do not necessarily represent those of their affiliated organizations, or those of the publisher, the editors and the reviewers. Any product that may be evaluated in this article, or claim that may be made by its manufacturer, is not guaranteed or endorsed by the publisher.

## Footnotes

1
https://en.wikipedia.org/wiki/Jin_dynasty

2
https://en.wikipedia.org/wiki/Qing_dynasty

3
https://reich.hms.harvard.edu/allen-ancient-dna-resource-aadr-downloadable-genotypes-present-day-and-ancient-dna-data

4
https://www.r-project.org/

5
https://www.phylotree.org/tree

6
https://isogg.org/

7
https://en.wikipedia.org/wiki/Northern_and_Southern_dynasties

8
https://en.wikipedia.org/wiki/Chuang_Guandong
